# Ecological Equivalence: A Realistic Assumption for Niche Theory as a Testable Alternative to Neutral Theory

**DOI:** 10.1371/journal.pone.0007460

**Published:** 2009-10-14

**Authors:** C. Patrick Doncaster

**Affiliations:** School of Biological Sciences, University of Southampton, Southampton, United Kingdom; University of Leeds, United Kingdom

## Abstract

**Background:**

Hubbell's 2001 neutral theory unifies biodiversity and biogeography by modelling steady-state distributions of species richness and abundances across spatio-temporal scales. Accurate predictions have issued from its core premise that all species have identical vital rates. Yet no ecologist believes that species are identical in reality. Here I explain this paradox in terms of the ecological equivalence that species must achieve at their coexistence equilibrium, defined by zero net fitness for all regardless of intrinsic differences between them. I show that the distinction of realised from intrinsic vital rates is crucial to evaluating community resilience.

**Principal Findings:**

An analysis of competitive interactions reveals how zero-sum patterns of abundance emerge for species with contrasting life-history traits as for identical species. I develop a stochastic model to simulate community assembly from a random drift of invasions sustaining the dynamics of recruitment following deaths and extinctions. Species are allocated identical intrinsic vital rates for neutral dynamics, or random intrinsic vital rates and competitive abilities for niche dynamics either on a continuous scale or between dominant-fugitive extremes. Resulting communities have steady-state distributions of the same type for more or less extremely differentiated species as for identical species. All produce negatively skewed log-normal distributions of species abundance, zero-sum relationships of total abundance to area, and Arrhenius relationships of species to area. Intrinsically identical species nevertheless support fewer total individuals, because their densities impact as strongly on each other as on themselves. Truly neutral communities have measurably lower abundance/area and higher species/abundance ratios.

**Conclusions:**

Neutral scenarios can be parameterized as null hypotheses for testing competitive release, which is a sure signal of niche dynamics. Ignoring the true strength of interactions between and within species risks a substantial misrepresentation of community resilience to habitat loss.

## Introduction

Hubbell's 2001 neutral theory (HNT) unifies the disciplines of biodiversity and biogeography by modelling steady-state distributions of species richness and relative species abundance across spatio-temporal scales [1]. Surprisingly accurate predictions have issued from its core premise that all species are exactly identical in their vital rates. As a null hypothesis to explain what should be observed if all species were perfectly equal with respect to all ecologically relevant properties, it has proved hard to refute [2]. Yet no ecologist, including Hubbell, believes that species are equivalent in reality [3], [4]. The challenge presented by HNT is to justify invoking anything more complex than ecological drift to define community structure [5]. Its extravagant simplicity has had an explosive impact on ecology (>1100 citations, rising exponentially), because it appears to discount 100 years of traditional conventions on niche differentiation. If biodiversity encompasses the great richness of differently attributed species that constitutes the natural world, how can ecological equivalence yield such predictive power about the numbers of species [6]? If HNT is based on a ludicrous assumption [7], then our conceptual understanding is thrown into disarray by its fit to empirical patterns [8]. Here I explain this paradox in terms of the ecological equivalence realised by coexisting species at demographic equilibrium. Analyses and simulations of coexistence equilibria demonstrate the emergent property of ecological equivalence amongst species with a rich diversity of attributes, leading to novel predictions for a quantifiable gradation in species-area relationships between neutral and niche models.

A neutral model of empirical relationships eliminates “the entire set of forces competing for a place in the explanation of the pattern” [9]. Accordingly, HNT assumes that all species behave identically in a zero-sum game such that the total density of individuals in a trophically similar community remains constant regardless of species composition. The defining image of this ecological equivalence is a tropical forest canopy, with remarkably constant total densities of trees regardless of large regional variations in constituent species [1]. Interpretations of zero-sum equivalence routinely omit to distinguish between the equal vital rates realized at the system carrying capacity approximated in this image (and most datasets), and the intrinsic vital rates that define the heritable character traits of each species. Models of HNT consistently prescribe identical intrinsic rates and niche dimensions. Hubbell [1] anticipated the disjuncture between realized and intrinsic rates by comparing ecological equivalence to the fitness invariance achieved at carrying capacity, allowing for different trade-off combinations in life-history traits. The prevailing convention, however, remains that ecological equivalence explicitly requires symmetric species with identical per capita vital rates, thereby promulgating the notion that HNT is built on an unrealistic foundation [3].

Theoretical studies have sought various ways to reconcile neutral patterns with niche concepts. Intrinsically similar species can coexist under niche theory [7], and niches add stabilizing mechanisms that are absent under the fitness equivalence of intrinsic neutrality [10]. Comparisons of niche to neutral simulations in a saturated system of fixed total abundance have shown that they can predict similar species-abundance distributions and species-area relationships [11], demonstrating that neutral patterns need not imply neutral processes [12]. Even neutral processes of intraspecific competition and dispersal limitation cannot be distinguished in principle for species-abundance predictions [13]–[16]. Here I use an analysis and simulation of Lotka-Volterra dynamics to model zero-sum ecological drift as an emergent property of stochastic niche structures at dynamic equilibrium. I explain its appearance in the steady-state distributions even of extremely dissimilar species in terms of the trivial expectation that species must achieve ecological equivalence at their coexistence equilibrium, which is defined by equal realised fitness for all. Although the predictions are standards of Lotka-Volterra analysis for a homogeneous environment, they drive a simulation that for the first time spans across dispersal-limited neutral to stochastic niche scenarios without fixing the total abundance of individuals.

The neutral simulation developed here is consistent with the models of Solé et al. [17] and Allouche & Kadmon [18] in having total species, *S*, abundance of individuals, *N*, and zero-sum dynamics as emergent properties (in contrast to refs [1], [11], [12], [19]). The *S* species are identical in all respects including interspecific interactions equal to intraspecific (in contrast to refs [13], [16]). Non-neutral simulations developed here extend the model of Chave et al. [11] by allowing competitive differences to vary stochastically on a continuous scale, as in Purves & Pacala [12]. They extend both these models by allowing pre-emptive recruitment and emergent zero-sum dynamics, and the model of Calcagno et al. [20] by adding dispersal limitation. They are consistent with Tilman's niche theory [21], [22] in their population abundances being a function of species-specific vital rates.

These simulations confirm the previously untested prediction [12] that colonization-competition trade-offs with stochastic colonization will exhibit zero-sum ecological drift and produce rank abundance curves that resemble neutral drift. Truly neutral dynamics should nevertheless sustain a lower total density of individuals at density-dependent equilibrium. This is because intrinsically identical species must interact as strongly between as within species. They therefore experience no competitive release in each others' presence, contrasting with the net release to larger populations obtained by segregated niches. The simulations demonstrate this fundamental difference, and I discuss its use as a signal for dynamic processes when predicting species-area relationships.

## Results

### Analysis of abundance patterns for two-niche communities

**Figure 1:**
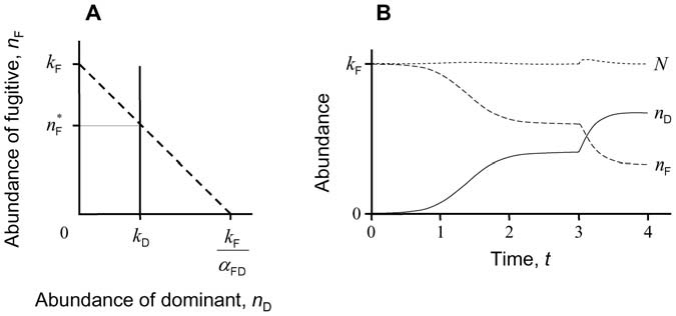
Equilibrium coexistence of a fugitive species invaded by a competitive dominant. With competition coefficients *α*_DF_ = 0, *α*_FD_ = 1, the fugitive persists provided it has the greater carrying capacity: *k*_F_ */k*_D_>1. (A) Lotka-Volterra phase plane with steady-state abundance at the intersection of the isoclines for the fugitive (dashed line) and the dominant (solid line). (B) Equilibration of abundances over time given by Runge-Kutta solutions to Equation 1, with a 20% drop in the dominant's intrinsic death rate, *d*_D_, imposed at *t* = 3 (equivalent to a rightward shift in its isocline) to illustrate the constancy of *N* = *n*_F_+*n*_D_.

Species characterized by extremely different intrinsic attributes can achieve ecological equivalence in a zero-sum game played out at dynamic equilibrium. Take for example a two-species community comprising a dominant competitor displacing the niche of a fugitive (e.g., [23]). The fugitive survives even under complete subordination, provided it trades competitive impact for faster growth capacity [24]. Figure 1 illustrates the equal fitness, zero-sum outcome at density-dependent equilibrium under this most extremely asymmetric competition. The carrying capacity of each species is a function of its intrinsic lifetime reproduction (detailed in Methods Equation 1), and equilibrium population sizes are therefore a function of the species-specific vital rates. Regardless of variation in the ratio of dominant to fugitive carrying capacities, 0≤*k*_D_ / *k*_F_≤1, the system density of individuals is attracted to the stable equilibrium at *N* = *n*_F_ + *n*_D_ = *k*_F_. Knocking out the fugitive reduces *N* to the smaller *k*_D_, but only until invasion by another fugitive. This may be expected to follow rapidly, given the fugitive characteristic of fast turnover. The steady-state scenario is effectively neutral by virtue of the dominant and fugitive realising identical vital rates and constant total density at their coexistence equilibrium despite contrasting intrinsic (heritable) rates. The reality that species differ in their life history traits therefore underpins the assumption of ecological equivalence, which then permits fitting of intrinsically neutral models with vital rates set equal to the realised rates. In the next section, these predictions are extended to simulate the drift of species invasions that sustains the dynamics of recruitment following deaths and extinctions amongst multiple species of dominants and fugitives.

**Figure 2:**
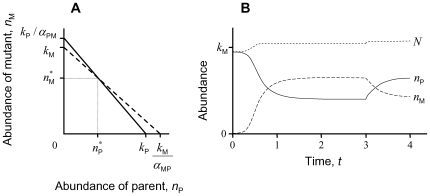
Equilibrium coexistence of a sexually reproducing parent population *n*_P_ invaded by an asexual mutant, *n*_M_. With the mutant having identical vital rates except for twice the intrinsic propagation rate per capita: *b*_M_ = 2⋅*b*_P_, the parent species persists if *α*_PM_<*k*_P_ */k*_M_. (A) Phase plane. (B) Equilibration of abundances over time given by Equation 1, with a 50% drop in the parent's intrinsic death rate imposed at *t* = 3 to illustrate approximate constancy of *N* = *n*_M_+*n*_P_.

The same principle of trade-offs in character traits conversely allows a sexually reproducing species to withstand invasion by highly fecund asexual mutants [25], [26]. A two-fold advantage to the mutant in growth capacity resulting from its production of female-only offspring is cancelled by even a small competitive edge for the parent species (Fig. 2). Sexual and asexual types coexist as ecological equivalents to the extent that each invades the other's population to symmetric (zero) net growth for all. Although the dynamics are not zero-sum if the mutant has some competitive impact on the parent species, they approach it the higher the impact of parent on mutant and the faster its growth capacity (albeit half the mutant's). Attributes such as these accommodate greater similarity between the types in their carrying capacities and competitive abilities, which aligns the two isoclines. A consequently reduced stability of the coexistence equilibrium may result in the sexual parent ousting the asexual mutant over time, for example if the latter accumulates deleterious mutations [26], [27].

These local-scale dynamics apply equally at the regional scale of biogeography, reconfiguring individual death as local extinction, and birth as habitat colonization [24]. Equally for regional as for local scales, rate equations take as many dimensions as species in the community, with their coupling together defining niche overlap [24], [28]. Coexistence of the species that make up a community is facilitated by their different heritable traits, which is a fundamental premise of niche theory. Ecological equivalence, and hence modelling by neutral theory is nevertheless possible by virtue of the coexistence equilibrium levelling the playing field to zero net growth for all.

The above examples of dominant versus fugitive and sexual versus asexual were illustrated with models that gave identical realised rates of both birth and death at coexistence equilibrium. Fitness invariance and zero-sum dynamics, however, require only that species have identical net rates of realised birth minus death. The simulations in the next section show how neutral-like dynamics are realised for communities of coexisting species with trade-offs in realized as well as intrinsic vital rates.

### Comparison of simulated neutral and multi-niche communities with drift

**Figure 3:**
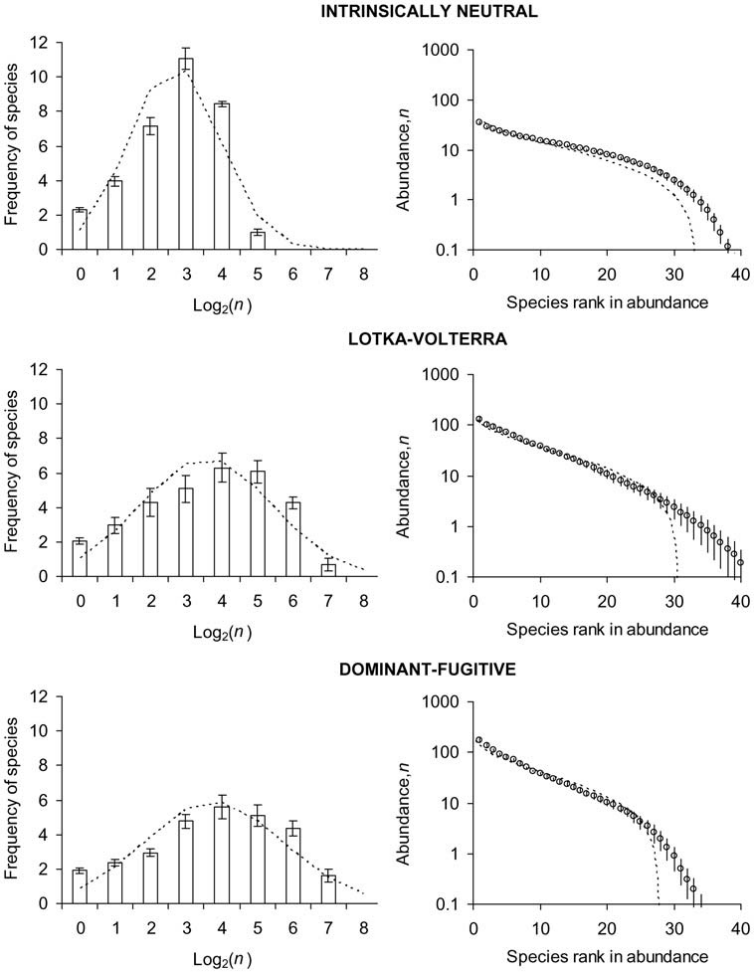
Simulated steady-states of species-abundance distributions (SADs). From top to bottom, graphs show average patterns for intrinsically neutral, Lotka-Volterra, and dominant-fugitive communities. SADs each show mean ± s.e. of six replicate communities with carrying capacity *K* = 1000 habitable patches. Frequencies are compared to log-normal (left-hand column) and MacArthur's broken-stick (right-hand column). See Methods for input parameter values and the process of random species assembly.

**Figure 4 pone-0007460-g004:**
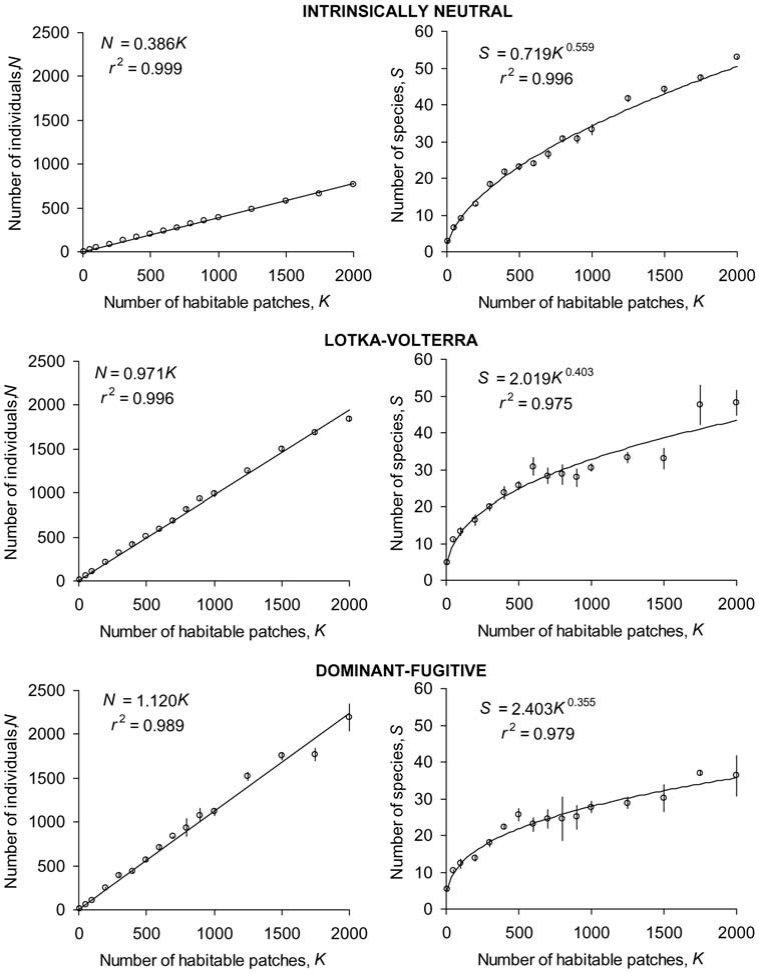
Simulated steady-states of species-area relationships (SARs). SARs each show mean ± s.e. of three replicate communities. See Methods for input parameter values and the process of random species assembly.

Figure 3 illustrates the species-abundance distributions and species-area relationships of randomly assembled *S*-species systems under drift of limited immigration and new-species invasions (protocols described in Simulation Methods). From top to bottom, its graphs show congruent patterns between an intrinsically neutral community with identical character traits for all species (equivalent to identically superimposed isoclines in Figs-1 and -2 models), and communities that trade growth capacity against competitive dominance increasingly starkly. The non-neutral communities sustain more total individuals and show greater spread in their responses, reflecting their variable life-history coefficients. Their communities nevertheless follow qualitatively the same patterns as those of neutral communities. For intrinsically neutral and niche-based communities alike, Fig. 3 shows species-abundance distributions negatively skewed from log-normal (all *P*<0.05, every *g*_1_<0), and an accelerating decline in rank abundances of rare species (cf. linear for Fisher log-series) that is significantly less precipitous than predicted by broken-stick models of randomly allocated abundances amongst fixed *S* and *N*; Fig. 4 shows constant densities of total individuals regardless of area (unambiguously linear), and Arrhenius relationships of species richness to area (unambiguously linear on log-log scales).

The extended tail of rare species seen in the Fig.-3 species-abundance distributions is caused by single-individual invaders replacing random extinctions of *n*-individual species. Further trials confirm that reduced dispersal limitation exacerbates the negative skew from the log-normal distribution, while sustaining a higher total density of individuals. The extinction-invasion imbalance sets the equilibrium species richness, *S*, as a power function of total population density, *N*. This can be expressed as the Arrhenius relationship: *S* = *cK^ z^* (Fig. 4 right-hand column) by virtue of the zero-sum relation of *N* to *K* (Fig. 4 left-hand column). Supporting Text S1 provides a full analysis of the departure from MacArthur's broken-stick model, and the derivations of the Arrhenius *c* and *z*. Further simulations show that reduced dispersal limitation raises *c* and reduces *z*, and a higher rate of new-species invasions raises *c* (though not *z*, in contrast to predictions from spatially explicit neutral models [29]).

The closely aligned proportionality of total individuals to habitable area for all communities illustrates emergent zero-sum dynamics for neutral and non-neutral scenarios (Fig. 4 left-hand column). Despite sharing this type of pattern, and rather similar densities of species (Fig. 4 right-hand column), the non-neutral communities sustain more than double the total individuals. This difference is caused by a more than halving of their competition coefficients on average (all *α_ij_* = 1 for neutral, mean *α_ij_* (*i*≠*j*) = 0.45 for Lotka-Volterra, mean ratio of 0∶1 values = 58∶42 for dominant-fugitive). The zero-sum gradient of *N* against *K* is simply the equilibrium fraction of occupied habitat, which is 1–1/*R* for a closed neutral scenario, where *R* is per capita lifetime reproduction before density regulation (*b/d* in Methods Equation 1 [23], [24]). The closed dominant-fugitive scenario modelled in Fig. 1 has a slope of *k*_F_ */K* = (1–1/*R*)/*α*, where *R* and *α* are system averages. Further simulation trials show the slope increasing with immigration, for example by a factor of 1.9 between closed and fully open (dispersal unlimited) Lotka-Volterra communities. Dispersal limitation therefore counterbalances effects of the net competitive release obtained in niche scenarios from *α_ij_*<1 (as also seen in models of heterogeneous environments [19]).

**Figure 5:**
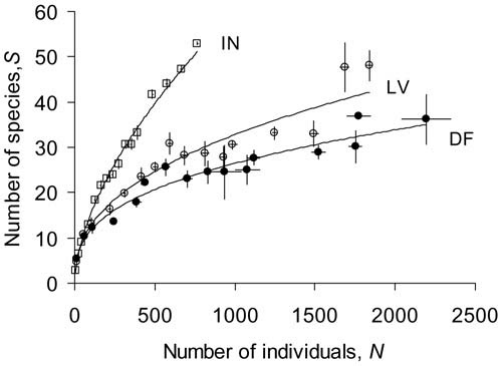
Simulated steady-state relationships of species to individuals. Each point shows the mean ± s.e. of the three replicate communities in Fig. 4, and regression lines on the means are the power functions for intrinsically neutral (top) Lotka-Volterra (middle) and dominant-fugitive (lower) scenarios.

The less crowded neutral scenario sustains a somewhat higher density of species than non-neutral scenarios (comparing Fig. 4 *z*-values for right-hand graphs), and consequently it maximizes species packing as expressed by the power function predicting *S* from *N* in Fig. 5. With no species intrinsically advantaged in the neutral scenario, its coefficient of power is higher than for pooled non-neutral scenarios (0.594 and 0.384 respectively, log-log covariate contrasts: *F*_1,42_ = 122.72, *P*<0.001). The lower coefficients of Lotka-Volterra and dominant-fugitive scenarios are further differentiated by competitive asymmetry (0.412 and 0.355 respectively, *F*_1,42_ = 7.24, *P*<0.01). In effect, the neutral scenario has the lowest average abundance of individuals per species, *n*, for a community of size *K* with given average *R*, which is also reflected in the modal values in Fig. 3 histograms for *K* = 1000 patches.

The lower *N* and *n* predicted for the intrinsically neutral scenario point to a detectable signal of steady-state intrinsically neutral dynamics: *α* = 1 for all, because intrinsically identical species cannot experience competitive release in each others' presence (cf. *α_ij_*<1 in niche models). These interactions may be measurable directly from field data as inter-specific impacts of equal magnitude to intra-specific impacts; alternatively, Lotka-Volterra models of the sort described here can estimate average competition coefficients at an observed equilibrium *N*, given an average *R* (a big proviso, as field data generally measure realised rather than intrinsic vital rates). This distinction of intrinsically neutral from non-neutral dynamics has been masked in previous theory by the convention for neutral models either to fix *N* [1], [11], [12] or to set zero interspecific impacts [13,16). By definition, identical species cannot be invisible to each other unless they are invisible to themselves, which would require density independent dynamics. Simulations of non-interacting species under density-dependent regulation therefore embody an extreme version of niche theory whereby each species occupies a unique niche, somehow completely differentiated by resource preferences rather than partially by trade-offs in vital rates. These models fit well to species abundance distributions in rainforests and coral reefs [13]–[16], though without providing any explanation for what attributes would allow each species to be invisible to all others (in contrast to the trade-off models). Indeed the condition is unrealistic at least for mature trees that partition a homogeneous environment by each making their own canopy. This so-called neutral scenario ([13], [16], more appositely a neutral-niche scenario) has no steady state outcomes in the analyses and simulations described here, because setting all *α_ij_* = 0 (*i*≠*j*) allows indefinite expansion of *S* and hence also of *N*. A slightly less extreme neutral-niche community is modelled by setting all interspecific impacts to a common low value. Simulations at *α_ij_* = 0.1 for all *i*≠*j* give a zero-sum relation *N* = 4.026*K*, which has >4-fold steeper gradient than that for the Lotka-Volterra scenario (Fig. 4) reflecting its >4-fold reduction in *α* and consistent with its representation of a highly niched scenario.

## Discussion

Although intrinsic identity is clearly not a necessary condition of ecological equivalence or of zero-sum abundances at dynamic equilibrium, only neutral models sustain these outcomes over all frequencies. It is their good fit to steady-state patterns of diversity and abundance even for communities subject to species turnover in ecological drift that has argued powerfully for niche differences having a limited role in community structure. The Fig.-3 simulations reveal these types of patterns to be equally well represented by niche models, however, despite constituent individuals and species achieving fitness equivalence only at dynamic equilibrium. Non-neutral dynamics of a mature community express the community-wide average of fluctuations either side of equilibrium. Outcomes regress to the equilibrium mean for a random assembly of species undergoing stochastic extinctions of rare members, regulated by spatially autocorrelated immigration, and replacement by initially rare invaders. The predicted power of neutral theory can be taken as evidence for ecological equivalence at the coexistence equilibrium of species with more or less different intrinsic attributes.

Modelling zero-sum ecological drift as an emergent property reveals a key distinguishing feature of truly neutral communities. Their intrinsically identical species self-regulate to a lower total density as a result of inter-specific impacts equalling intra-specific impacts. Any empirical test for competitive release is therefore also a test for niche structure. For example, removing habitat is predicted to give a relative or absolute advantage to species towards the fugitive end of a dominant-fugitive spectrum, which may be picked up in correlated life-history traits for winners or losers under habitat loss or degradation [23], [24]. In contrast, neutral dynamics lead to sudden biodiversity collapse at a system-wide extinction threshold of habitat [17]. The extinction threshold of habitat for a resource-limited metapopulation is set by the fraction 1/*R* [30], [31]. The value of *R* is thus an important yardstick of resilience in conservation planning. A neutral model fitted to empirical zero-sum abundances will overestimate their community-wide *R*, and hence overestimate community resilience, if *α_ij_* are overvalued by setting all to unity. Likewise, a neutral model that sets all *α_ij_* = 0 (*i*≠*j*) will underestimate *R*, and hence resilience, if the *α_ij_* are undervalued by setting all to zero.

Ecological equivalence is a much more permissive requirement for neutrality than is currently acknowledged in theoretical developments on HNT. Coexistence equilibria largely achieve the neutrality-defining mission, to eliminate all of the forces competing for a place in explanations of pattern. It remains an open question whether they do so best amongst species with most or least competitive release in each others' presence (e.g., Fig. 1 versus Fig. 2 respectively, and Fig. 3 dominant-fugitive versus Lotka-Volterra respectively; [7], [10], [32]). Models need to incorporate the ecologically realistic dynamics of interspecific interactions simulated here in order to explore the true nature of competitive release between extreme scenarios of niches that are all intrinsically identical (HNT [1]) and intrinsically unique [13], [16]. Simulations of niches distributed along environmental gradients have found emerging groups of intrinsically similar species over evolutionary timescales [33]. For the spatially homogeneous environments modelled here, competition-recruitment trade-offs will always sustain species differences. In their absence, however, homogenous environments will tend to favour fast-recruiting competitive dominants. This species type may eventually prevail, with runaway selection checked by other forces such as predation, disease, mutation accumulation and environmental variability. These systems would merit further study because many of their attributes could be those of intrinsically neutral dynamics.

## Methods

The following protocols apply to simulations of single and multi-niche communities with density-dependent recruitment and density-independent loss of individuals. They produce the outcomes illustrated in Figs 3–5 from input parameters specified at the end of this Section. The general model has species-specific vital rates; the intrinsically neutral and dominant-fugitive scenarios are special cases of this model, with constrained parameter values.

The community occupies a homogenous environment represented by a matrix of *K* equally accessible habitat patches within a wider meta-community of *K_m_* patches. The dynamics of individual births and deaths are modelled at each time step by species-specific probability *b* of each resident, immigrant, and individual of new invading species producing a propagule, and species-specific probability *d* of death for each patch resident. Recruitment to a patch is more or less suppressed from intrinsic rate *b* by the presence there of other species according to the value of *α_ij_*, the impact of species *j* on species *i* relative to *i* on itself, where the intraspecific impact *α_ii_* = 1 always. A patch can be occupied by only one individual of a species, and by only one species unless all its resident *α_ij_*<1. Conventional Lotka-Volterra competition is thus set in a metapopulation context by equating individual births and deaths to local colonisations and extinctions (following [24], consistent with [20]). A large closed metapopulation comprising *S* species has rates of change for each species *i* in its abundance *n_i_* of individuals (or equally of occupied patches) over time *t* approximated by:
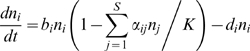
(1)This is the rate equation that also drives the dynamics of Figs 1 and 2, where *k_i_* = (1–*d_i_/b_i_*)*K*. Coexistence of any two species to positive equilibrium *n*_1_, *n*_2_ requires them to have intrinsic differences such that *k*_1_>*α*_12_ *k*_2_ and *k*_2_>*α*_21_ *k*_1_.

Each time-step in the simulation offers an opportunity for one individual of each of two new species to attempt invasion (regardless of the size of the meta-community). Each new species *i* has randomly set competitive impacts with respect to each other resident species *j*, of *α_ij_* received and *α_ji_* imposed. It has randomly set *b_i_*, and an intrinsic lifetime reproduction *R_i_* = *b_i_/d_i_* that is stratified in direct proportion to its dominance rank amongst residents, obtained from its ranked mean *α*-received minus mean *α*-imposed. For example, an invader with higher dominance than all of three resident species will have random *R_i_* stratified in the bottom quartile of set limits *R*_min_ to *R*_max_. Communities are thereby structured on a stochastic life-history trade-off between competitive dominance and population growth capacity. This competition-growth trade-off is a well-established feature of many real communities, which captures the fundamental life-history principle of costly adaptations [11], [17], [21]. Its effect on the community is to prevent escalations of growth capacity or competitive dominance amongst the invading species. Neutral communities are a special case, with identical values of *b* and *R* for all species and *α* = 1 for all.

At each time step, new invaders and every resident each have species-specific probability *b* of producing a propagule. Each propagule has small probability *ν* of speciation (following [1]). The sample community additionally receives immigrant propagules of its resident species that arrive from the wider meta-community in proportion to their expected numbers out there ([*K_m_/K*–1]*n_i_*), assuming the same density *n_i_*/*K* of each species *i* as in the sample community, and in proportion to their probability (*K/K_m_*) of landing within the sample community, and modified by a dispersal limitation parameter *ω*. In effect, for each resident species in the community, [(1–*K/K_m_*) *n_i_*]^1–*ω*^ external residents each produce an immigrating propagule with probability *b_i_*. Thus if *K_m_*≫*K* and *ω* = 0, a colonist is just as likely to be an immigrant from outside as produced from within the sample community (no dispersal limitation, following [1]). This likelihood reduces for *ω*>0, and also for smaller *K_m_*. None of the propagules generated within the sample community emigrate out into the meta-community, making *K* a sink if smaller than *K_m_* (sensu [34]), or a closed community if equal to *K_m_*. The simulation is thus conceptually equivalent to randomly assembled *S*-species systems previously studied (e.g., [35]), except that it additionally accommodates a random drift of invasions to sustain the dynamics of recruitment following deaths and extinctions.

Each propagule lands on a random patch within the sample community and establishes there only if (a) its species is not already present, and (b) it beats each probability *α_ij_* of repulsion by each other resident species *j*, and (c) it either beats the odds on repulsion by all other propagules simultaneously attempting to colonise the patch, or benefits from the random chance of being the first arrival amongst them. Each pre-established resident risks death with species-specific probability *d_i_* = *b_i_/R_i_* at each time step. Each patch has probability *X* of a catastrophic hazard at each time step that extirpates all its occupants. The model thus captures the principles of stochastic niche theory [21], [22] and pre-emptive advantage [20].

Each of the replicate communities contributing to distributions and relationships in Figs 3–5 is represented by values averaged over time-steps 401–500, long after the asymptote of species richness. For all graphs in Figs 3–5, meta-community carrying capacity *K_m_* = 10^6^, dispersal limitation parameter *ω* = 0.5, speciation probability per resident propagation event *ν* = 10^−12^, two invasion attempts per time-step (setting Hubbell's [1] fundamental biodiversity number *θ*∼4 independently of *K_m_*), probability of catastrophe per patch *X* = 0.01. For neutral communities, all species take competition coefficients *α* = 1, individual intrinsic propagation probability *b* = 0.5, individual intrinsic lifetime reproduction *R* = 1.5 (so lifespan *R/b* = 3); for Lotka-Volterra communities, each species *i* takes random 0≤*α_ij_*≤1, random 0≤*b_i_*≤1, *R_i_* between 1.2 and 1.8 and proportional to dominance rank; dominant-fugitive communities are as Lotka-Volterra except for random binary *α_ij_* = 0 or 1. All scenarios are thereby sampled from a large meta-community with moderate dispersal limitation, low extrinsic mortality, and sufficient invasions to sustain a reasonably high asymptote of species richness from the starting point of two species each occupying 5 patches. Skew in the lognormal distribution of species abundances (Fig. 3) was measured for each replicate in its dimensionless third moment about the mean, *g*_1_ [36], and confidence limits for the sample of six values were tested against *H*_0_: *g*_1_ = 0.

## Supporting Information

Text S1 Analysis of neutral community assembly
(0.06 MB DOC)
