# Supplementary material for: Ecological Equivalence: A Realistic Assumption for Niche Theory as a Testable Alternative to Neutral Theory
Source: PLoS One. 2009 Oct 14;4(10):e7460. doi: 10.1371/journal.pone.0007460 (PMC2759543; doi:10.1371/journal.pone.0007460)
Supplement: Text S1 — Analysis of neutral community assembly (0.06 MB DOC) [file pone.0007460.s001.doc]

### Supporting Information: Analysis of neutral community assembly

Here we derive analytically the frequency distribution of species abundances expected for the simulated neutral dynamics under drift. Consider a community of *N* identical individuals each belonging to one of *S* identical species. In the absence of immigration and extrinsic mortality, the equilibrium *N* is given by setting *dN/dt* = 0 in main-text Equation 1 to obtain:

*N* = *K*(1 – *d/b*) (S1)

We wish to find the associated equilibrium value of *S*. If births and deaths have randomised the species abundances within a closed population of fixed total *N*, their equilibria are represented by the lengths of each part of a stick that has been broken at *S* – 1 random points along its *N*-unit length [1,2]. This ‘broken-stick’ model predicts *S*  *N*^0.5^ [3]. However, its rank-abundance distribution does not fit the simulation output (Fig. 3 top right-hand graph) because it takes no account of species extinctions and invasions.

To account for an extinction-invasion balance, we represent the community of 1-, 2-, … *n*-individual species by a necklace strung in random order with *S* white beads and *N* – *S* black beads. Clockwise around the necklace, each white bead represents the first individual of a species. Two consecutive white beads, w-w, therefore indicate a 1-individual species. The segment w-w-b-b-w-b-w indicates a 1-individual, a 3-individual. and a 2-individual species, and so on. Assume the randomisation of species abundances is sustained by random walks of births filling spaces left by deaths, without disturbing the equilibrium total of *N* individuals. In the first instance, we assume also that any random extinction of an *n*-individual species is compensated by the arrival of an equally abundant invader. The probability of obtaining a 1-individual species from any pair of abutting beads (i.e., w-w) is then (*S/N*)(*S/N*), so the expected frequency of 1-individual species is *N*(*S/N*)^2^; likewise the frequency of 2-individual species (w-b-w) is *N*(*S/N*)^2^(1 – *S/N*). Generally, the frequency of *n*-individual species is:

*f*(*n*) = (*S*^2^*/N*)(1 – *S/N*)*^n^*^–1^ (S2)

which declines monotonically from *n* = 1 to *N* – *S*, with Σ*f*(*n*) = *S* and Σ*n⋅f*(*n*) = *N*.

We now impose the reality from the simulation that invaders arrive as 1-individual species to replace extinctions of *n*-individual species. Each time step allows two attempted invasions each of a single individual of a new species. At the dynamic equilibrium of births matching deaths, each invader has probability *d* of establishing, where *d* is the per capita death rate in Equation 1. The expected invasion rate is therefore 2*d*. For established residents, the extinction probability of a *n*-individual species is [*d*(1 – *d*)]*^n^*. This is the chance that every one of its *n* individuals dies without propagating successfully, which diminishes rapidly with *n* (i.e., rarer species are much more prone to extinction). The corresponding extinction rate is not straightforward to model because it simultaneously depends on, and influences, the frequency distribution of *n*-individual species. We can at least predict boundary conditions.

The lower boundary of *f*(*n*) is given by the frequency distribution of *n*-individual species that results from *t* rounds of extinction before any invasion. Thus for *n* = 1 to *N* – *S*:

*f*(*n*) = (*S*^2^*/N*)(1 – *S/N*)*^n^*^–1^[1 – (*d*(1 – *d*))*^n^*]*^t^* (S3)

The community sustains a constant *S* species if the compound sum of extinctions over *t* time-steps just matches the total 2⋅*t*⋅*d* invasions expected after the same *t* steps. An iterative solution can be obtained for *t* such that *S* – Σ*f*(*n*) = 2*⋅t*⋅*d*. The corresponding upper bound of *f*(*n*) is given by setting *t* = 0 to obtain Equation S2, which is realised only if each invading species immediately expands in abundance to fill the space left by each extinction of a *n*-individual species. As expected, the observed distribution of *n*-individual species from the simulation lies midway between these boundaries (Fig. S1).

**A**

**B**

**Figure S1. Frequency distributions of equilibrium species abundances with fast/slow replacement of extinctions.** Observed ± s.e. frequencies from six replicate simulations (grey bars and vertical lines), and expected if each *n*-individual extinction is immediately replaced by a *n*-individual invasion (blue), or expected if *t* = 7.36 rounds of extinction occur before 2⋅*t⋅d* invasions of 1-individual species (red). (**A**) *K* = 1000; (**B**) *K* = 500. Other parameters as for intrinsically neutral simulations in main-text Figs 3-4, except *X* = 0, *ω* = 1.

The good fit of the upper boundary in Fig. S1 for all but the rarest species sustains the negative skew in the log-normal distribution of Fig. 3 top left-hand graph. The higher susceptibility of rarer species to extinction nevertheless pulls down the frequencies of 1- and 2-individual species in Fig. S1 halfway towards the lower boundary, causing the rank-abundance distribution to rise above broken-stick expectation in Fig. 3 top right-hand graph.

At both upper and lower boundaries, the frequency of 1-individual species, *f*(1), is a constant per unit area, set only by the character traits of the species (and consequently invariant across Fig. S1 A-B). For example, in the neutral scenario of identical species, 1/*f*(1) is the minimum area required for each individual to just replace itself on average (see for example [3]). Putting Equation S1 into S3 at *n* = 1 gives the Arrhenius relation:

$S=c\cdot K^{z}\text{,} \text{where} z=0.5 \text{and} c=\left[\frac{f\left(1\right)\cdot\left(1-d/b\right)}{\left[1-d\cdot\left(1-d\right)\right]^{ t}}\right]^{0.5}$ (S4)

Simulations described in the main text show that the values of *z* and *c* depend on other life-history parameters not considered in this analysis, including species-specific values *R_i_*, *b_i_*, *α_ij_* characteristic of non-neutral scenarios, and *X* > 0, *ω* < 1, and faster invasion rate.

### References

1. MacArthur RH (1957) On the relative abundance of bird species. Proc Natl Acad Sci U S A 43: 293-295.
2. MacArthur RH (1960) On the relative abundance of species. Am Nat 94: 25-36.
3. May RM (1975) Patterns of species abundance and diversity. In: Cody ML, Diamond JM, editors. Ecology and evolution of communities. Cambridge, Mass: Harvard University Press. pp. 81-120.
